# Evaluation of complications and quality of life of patient after surgical extraction of mandibular impacted third molar teeth

**DOI:** 10.1186/s12903-024-03877-8

**Published:** 2024-01-25

**Authors:** Gunay Gojayeva, Gorkem Tekin, Nesrin Saruhan Kose, Omur Dereci, Yasin Caglar Kosar, Gizem Caliskan

**Affiliations:** 1 Specialist in Oral and Maxillofacial Surgeon, Private Practice, Eskişehir, Turkey; 2 Department of Oral and Maxillofacial Surgery, Faculty of Dentistry, Eskisehir Osmangazi University, Eskisehir, Turkey

**Keywords:** Impacted tooth, Complications, Health Quality

## Abstract

**Background:**

The aim of our study is to evaluate the postoperative complications after the extraction of impacted third molar teeth and to investigate the effects of these complications on the quality of life of patients.

**Methods:**

Demographic, clinical, and radiological evaluations were conducted, covering factors like age, gender, and tooth position. Clinical measurements, pain and edema assessments, and quality of life evaluations through OHIP-14 scores were performed. Preoperative and postoperative mouth opening, trismus, alveolitis and dehiscence were evaluated.

**Results:**

A total of 100 patients were included in our study. No significant gender-based differences were found in measurements, pain, or swelling. There was no statistically significant difference between the preoperative and postoperative results of difference A-C, difference B-E, difference A-D, and difference mouth opening. Procedure duration correlated positively with age, alveolar osteitis, trismus, and swelling. Postoperative quality of life, assessed by OHIP-14, demonstrated a negative correlation with age and trismus. It was observed that the gender and the tooth positions of the patients had no effect on the severity of postoperative pain and edema.

**Conclusions:**

As the age of the patients increases and the duration of the procedure increases, the rate of postoperative complications increases and it is concluded that the quality of life decreases significantly.

## Background

Although impacted tooth extraction is the most frequently performed surgical procedure in maxillofacial surgery, the incidence of complications is also one of the highest. Not all impacted teeth can cause problems, but all have this potential [1]. Some of these teeth are indicated for therapeutic purposes and the other for prophylactic extraction indications. Therapeutic causes include pericoronitis, caries, periodontal diseases, presence of pathology such as cyst or tumor, tooth extraction for orthodontic and prosthetic treatment. Extraction of impacted teeth for prophylactic purposes is recommended for patients at risk of infection and those dealing with risky sports fields [2, 3].

Studies have shown that there is a 10% incidence of complications after surgical extraction of mandibular impacted third molars [4]. These complications can be seen as expected and predictable such as swelling and pain and more serious complications such as mandible fracture [5]. The overall incidence and severity of complications are directly related to the depth of the impaction, medical history, gender, age, oral contraceptives, presence of pericoronitis, smoking, poor oral hygiene, relationship of third molar to the inferior alveolar nerve, surgical time, surgical technique, surgeon experience, use of perioperative antibiotics, use of topical antiseptics, use of intra-socket medications, and anesthetic technique. The most common complications in mandibular impacted third molar surgery are postoperative infection, edema, trismus, hemorrhage, paresthesia, and mandibular fractures [6–8].

Postoperative edema, pain and trismus affect the lives of patients not only functionally but also socially [9]. The quality of life of patients is a multifactorial concept and quality of life scales have made significant progress in the last 10 years. The concept of oral health-related quality of life (OHIP-14) refers to the impact of oral health conditions on daily activities, quality of life, and an individual’s health. OHIP-14 consists of 14 questions and 5 answers scored between 0 and 4 that can be given to each question. Evaluation in OHIP 14 is made on 8 data separately under 7 main headings and the sum of all these category scores. High scores indicate a negative impact on quality of life, and low scores indicate a positive impact on quality of life [10, 11].

The aim of our study is to evaluate the complications that occur after mandibular impacted third molar surgery, to evaluate the relationship between these complications and the age, gender, position of the teeth, and the duration of the procedure, and to determine how the postoperative complications affect the quality of life of the patients.

## Material and method

### Sample

This retrospective study was approved by the Osmangazi University Non-Interventional Clinical Research Ethics Committee dated 26.01.2021 and numbered 2021-39. Patients with uncontrolled systemic diseases (renal, diabetes, hypertension etc.), pregnant individuals, those suspected of being pregnant, patients using immunosuppressive drugs, individuals with syndromes, those with incomplete medical records (patients who did not follow the postoperative assessment schedule), and patients without records of impacted tooth panoramic radiography were excluded from the study. Preoperative demographic, clinical and radiological evaluations of the patients were performed retrospectively. In demographic evaluation made retrospectively; age, gender, systemic diseases, and educational status of the patients were evaluated.

### Measurements

In clinical evaluation; number of extracted tooth, duration of procedure, preoperative mouth opening (mm), postoperative mouth opening (mm), difference between postoperative mouth opening and preoperative mouth opening (mm), preoperative A-C length (mm), preoperative B-E length (mm), preoperative A-D length (mm), postoperative A-C length (mm), postoperative B-E length (mm), postoperative A-D length (mm), the difference between A-C, B-E and A-D lengths postoperatively and preoperatively (mm), alveolar osteitis, dehiscence, postoperative pain and swelling on the 1st 3rd and 7th days, OHIP-14 data were measured, retrospectively. In retrospective radiological evaluation; positions of the mandibular impacted third molar teeth were determined. Surface areas were taken with a flexible ruler while the patient was sitting upright and the mandible was in physiological resting position. In order to determine the mouth opening, taking the mesial corners of the first permanent incisors in the upper and lower jaw as reference, the distance at the maximum opening movement was calculated with the help of a ruler; The patient was measured immediately before surgery and on the 3rd and 7th postoperative days and recorded in cm.

Swelling was evaluated using the following signs:

A-C length: The distance from the posterior point of the tragus to the corner of the mouth.

B-E length: The distance from the lateral canthus to the lowest point of the angulus.

A-D length: Distance from posterior point of tragus to pogonium.

VRS (0,1…,5) was used to evaluate pain and swelling. A score of 0 represented no pain and 5 represented excessive pain. Patients completed this scale on the 1st, 3rd and 7th postoperative days. The OHIP-14 form was used to evaluate the quality of life of the patients. The diagnosis of alveolar osteitis was made in patients who present with symptoms such as pain between the 2nd and 4th days after extraction, tenderness during probing, an empty socket, and the complaint of food debris in the extraction socket.

### Statistical analysis

For statistical analysis, IBM SPSS Statistics Version 22 package program (Armonk, NY: IBM Corp.) was used. Evaluation of the normal distribution of the data was done with the Shapiro Wilk’s test. Independent samples t-test and Mann-Whitney U Test were used to compare subgroups. Pearson’s correlation analysis was used to evaluate relationships between variables. The p value < 0.05 was accepted as significant.

## Results

A total of 100 patients who applied to Eskişehir Osmangazi University Faculty of Dentistry, Department of Oral and Maxillofacial Surgery with the complaint of mandibular impacted third molars were included in our study. Our study; It was performed in 100 patients aged between 16 and 53 (mean 25.91 ± 7.63), 36 males (36%), 64 females (64%) with mandibular impacted third molars. When the patients are evaluated in terms of their educational status; 9 (9%) of the patients were primary school graduates, 39 (39%) secondary school graduates, and 52 (52%) university graduates. When evaluated in terms of systemic diseases; it was observed that 95 of the patients (95%) did not have any systemic disease, 3 of them had controlled hypothyroidism (3%), and 2 of them (2%) had controlled diabetes.

Of the impacted third molars, 50 (50%) were located in the right mandible, and 50 (50%) were in the left mandible. When the impacted third molars are classified according to their positions; It was observed that 36 (36%) were vertical, 23 (23%) mesioangular, 20 (20%) distoangular, 19 (19%) horizontal, 2 (2%) buccolingual. Descriptive statistics results of the data are shown in Table 1.

**Table 1:** Descriptive statistics of numerical data

	Min.	Max.	Mean	Median	SD
Age	16	53	25.91	23	7.62
Surgery time	4	60	20.01	18	11.05
Pre-op A-C	97	142	114.41	114.5	8.06
Pre-op B-E	79	156	102.02	101	10.66
Pre-op A-D	120	177	148.42	148	10.57
Post-op A-C	104	144	118.68	117	7.86
Post-op B-E	89	162	107.53	106	10.20
Post-op A-D	125	190	154.68	152	11.23
Difference A-C	0	15	4.27	3	3.29
Difference B-E	0	24	5.51	4	4.90
Difference A-D	0	39	6.26	5	5.46
Pre-op mouth opening	29	64	47.16	47	6.06
Post-op mouth opening	23	54	40.63	42	7.10
Difference mouth opening	0	26	6.53	4.5	6.18
OHIP-14 Total Score	0	36	13.3	13	8.19

Min: Minimum

Max: Maximum

SD: Standard Deviation

Postoperative evaluation revealed alveolar osteitis in 13 (13%) patients and dehiscence in 6 patients (6%).

Post-operatively, the values of pain and swelling on the 1st, 3rd and 7th days are given in Table 2. On the first postoperative day, 7% of the patients had no pain and only 8% had severe pain. It was concluded that the pain was completely relieved in 74% of the patients on the 7th postoperative day.

**Table 2:** Postoperative pain and swelling assessments

	Day 1 n (%)	Day 3 n (%)	Day 7 n (%)
No Pain (0)	7 (7%)	29 (29%)	74 (74%)
Mild Pain (1)	27 (27%)	36 (36%)	18 (18%)
Moderate Pain (2)	21 (21%)	18 (18%)	3 (3%)
Severe Pain (3)	23 (23%)	12 (12%)	3 (3%)
Very Severe (4)	14 (14%)	5 (5%)	2 (2%)
Excessive Pain(5)	8 (8%)	0 (0%)	0 (0%)
No Swelling(0)	7 (7%)	13 (13%)	60 (60%)
Mild Swelling(1)	22 (22%)	26 (26%)	33 (33%)
Moderate Swelling(2)	28 (28%)	25 (25%)	6 (6%)
Severe Swelling(3)	24 (24%)	22 (22%)	1 (1%)
Very severe swelling(4)	4 (4%)	5 (5%)	0 (0%)
Excessive Swelling(5)	15 (15%)	9 (9%)	0 (0%)

When comparative statistics are made in terms of gender; There was no statistically significant difference between men and women in terms of difference A-C, B-E and A-D values, difference mouth opening, OHIP-14 scores, age, duration of procedure, pain and swelling values between men and women (*p* > 0.05)(Table 3).

**Table 3:** Comparative statistics by gender

	Female min-max (mean. ± ss)	Male min-max (mean. ± ss)	P
Age	17–44 (25.27 ± 6.96)	16–53 (27.06 ± 8.66)	0.31
Surgery time	4–51 (19.63 ± 10.25)	7–60 (20.69 ± 12.45)	0.96
Difference A-C	0–13 (4.56 ± 3.45)	0–15 (3.75 ± 2.97)	0.26
Difference B-E	0–24 (5.98 ± 5.27)	0–19 (4.67 ± 4.09)	0.35
Difference A-D	0–39 (6.19 ± 5.72)	0–18 (6.39 ± 5.02)	0.82
Difference mouth opening	0–24 (6.98 ± 6.38)	0–26 (5.72 ± 5.80)	0.37
OHIP-14	0–36 (12.50 ± 7.90)	0–36 (14.72 ± 8.62)	0.25
Pain Total Score	0–12 (3.89 ± 2.66)	0–13 (4.28 ± 3.09)	0.65
Swelling Total Score	0–11 (4.81 ± 3.05)	0–13 (5.22 ± 3.01)	0.50

Min: Minimum

Max: Maximum

SD: Standard Deviation

When the impacted mandibular third molars are evaluated in terms of their positions; The descriptive values of the total scores of age, procedure time, difference A-C, B-E and A-D, difference mouth opening, OHIP-14 scores, pain and swelling values are shown in Table 4.

**Table 4:** Evaluation in terms of tooth positions

	Mesioangular min-max(mean ± sd)	Vertical min-max(mean ± sd)	Horisontal min-max(mean ± sd)	Distoangular min-max(mean ± sd)	Buccolingual min-max(mean ± sd)
Age	16–41 (22.78 ± 6.03)	18–53 (25.94 ± 8,36)	17–45 (28,16 ± 7.81)	19–44 (27.8 ± 7.11)	18–24 (21 ± 4.24)
Surgery time	5–45 (21.26 ± 9.07)	5–51 (17.14 ± 10.76)	10–60 (26.21 ± 11.98)	4–35 (15.85 ± 8.6)	40–40 (40.00 ± 0.00)
Difference A-C	0–12 (3.83 ± 3.29)	1–13 (5.08 ± 3.13)	0–11 (3.58 ± 2.89)	0–15 (4.15 ± 3.93)	2–3 (2.5 ± 0.71)
Difference B-E	0–24 (7.39 ± 6.50)	0–18 (5.69 ± 4.96)	0–12 (3.79 ± 3.34)	0–13 (4.55 ± 3.45)	6–7 (6.50 ± 0.71)
Difference A-D	0–16 (4.83 ± 3.91)	0–17 (6 ± 4.96)	0–13 (6.95 ± 3.92)	1–39 (8.25 ± 8.65)	1–1 (1.0 ± 0.00)
Difference mouth opening	0–24 (5.48 ± 5.99)	0–26 (5.50 ± 5.59)	0–24 (8.47 ± 6.84)	0–22 (7.80 ± 6.8)	3–9 (6.00 ± 4.24)
OHIP-14 Total score	1–36 (15.09 ± 11.21)	0–26 (13,25 ± 6.99)	1–22 (13.37 ± 6.1)	0–28 (10.90 ± 7.92)	10–24 (17.0 ± 9.89)
Total pain	0–13 (3.83 ± 2.82)	0–12 (3.78 ± 3.13)	0–10 (4.58 ± 2.87)	0–8 (4.0 ± 2.20)	4–8 (6.0 ± 2.82)
Total swelling	0–11 (4.22 ± 3.08)	0–13 (4.92 ± 3.30)	0–11 (5.26 ± 3.46)	0–10 (5.80 ± 2.61)	3–3 (3.0 ± 0.00)

Min: Minimum

Max: Maximum

SD: Standard Deviation

The results of the correlation test, which we performed to determine the relationships between the variables, are shown in Table 5.

**Table 5:** Correlation values of variables

		1	2	3	4		5		6		7		8		9		10		11		12	
1	Age	1																				
2	Gender	-0,101	1																			
3	Tooth pozition	,294**	-0,12	1																		
4	Surgical time	0,019	0,004	-0,025	1																	
5	Difference mouth opening	-0,142	0,089	0,177	,305**		1															
6	Alveolar Osteitis	0,185	0,042	-0,033	,236*		0,126		1													
7	OHIP14Total score	-,213*	-0,115	-0,089	0,134		,230*		0,062		1											
8	Total pain	0,065	-0,044	0,118	-0,068		0,092		-0,116		0,105		1									
9	Total swelling	0,028	-0,066	0,19	-0,126		0,045		-0,115		0,003		,345**		1							
10	Difference AC	-0,052	0,112	-0,072	-0,09		0,09		-,246*		0,033		0,097		0,023		1					
11	Difference BE	-,202*	0,094	-0,137	0,107		0,121		-0,076		0,109		-0,104		-0,168		,217*		1			
12	Difference AD	0,157	-0,022	0,127	-0,07		0,146		0,021		0,105		0,043		-0,018		,272**		0,11		1	

**. Correlation is significant at the 0.01 level (2-tailed).

*. Correlation is significant at the 0.05 level (2-tailed).

## Discussion

Edema, pain, trismus, bleeding, alveolar osteitis and paresthesia are the most common complications after mandibular impacted third molar surgery. Damage to the second molar tooth, infection and mandible fractures are less common complications [12].

Some of the studies have argued that there is a significant relationship between the age of the patients and the complications. This relationship is attributed to the fact that as the age of the patients increases, more procedures are performed due to the increased bone density, the duration of the operation is prolonged, the root formation is completed with increasing age and the healing capacity decreases [13, 14]. In our study, it was observed that there was a positive correlation between the age of the patients and the duration of the procedure, tooth position, alveolar osteitis, total pain, total swelling, difference AD values, and a negative correlation between difference mouth opening, OHIP 14 total score, difference AC, difference BE values. However, among these values, only tooth position (*p* = 003), OHIP-14 total score (*p* = 0.033) and difference BE (*p* = 0.044) were found to be statistically significantly different from patient age.

Capuzzi et al. [15] reported that male patients had more pain complaints postoperatively. Monaco et al. [16] reported that gender had a statistically significant effect on postoperative pain and edema. They found that the incidence of postoperative edema (12.7%) in female patients was statistically significantly higher than in male patients (1.4%). In our study, it was observed that the incidence of postoperative pain and edema was similar between female and male, and there was no statistically significant difference between them.

Prolongation of the procedure time is shown as one of the important factors in the development of postoperative complications [17]. Pedersen [18] argues that the severity of postoperative pain is related to the prolongation of the operation time, while postoperative edema and trismus are not. Benediktsdottir et al. [7] found that the operation times of mandibular impacted third molars in a horizontal position were statistically significantly higher than those in a vertical position. It was observed that there was a positive correlation between the duration of the procedure and age, gender, difference in mouth opening, alveolar osteitis, OHIP-14 total score, difference BE values, and a negative correlation between tooth position, pain total, swelling total, difference AC, difference AD values. It was observed that the incidence of alveolar osteitis and trismus increased statistically as the duration of the procedure increased.

Various methods have been proposed in the literature for the evaluation of postoperative facial edema. These are computed tomography (CT), magnetic resonance imaging (MRI), ultrasonography (USG), measurement with laser, measurement with flexible ruler, and evaluation on a questionnaire [19]. In our study, the measurement method with a flexible ruler was used.

Kim et al. [20] measured the distances from the corner of the mouth to the tragus and from the soft tissue pogonion to the tragus to assess postoperative swelling. The measured distance was recorded preoperatively, postoperative day 1 and day 7. The difference between pre- and post-operative measurements was calculated. The elderly group (over 30 years) has been shown to have a significantly higher swelling rate than the younger group (under 30 years) (P 0.038). Brucoli et al. [21] after surgery, patients assessed swelling using a tape measure and took three measurements using five reference points: corner of the eye/angle of the lower jaw; tragus/corner of the mouth; tragus/pogonion. No significant difference was found between genders in post-operative measurements. In our study, preoperative, 1st day, 3rd day and 7th day (A - C), (B - E) and (A - D) distances were measured to evaluate postoperative swelling. To determine the amount of swelling after extraction of mandibular impacted third molars and also to investigate which of the upper, middle and lower 1/3 parts of the face was more affected, the difference of each measurement (e.g., A-C) on days 1, 3 and 7 was calculated. When we look at the preoperative and postoperative measurements, the highest difference was recorded as 0–39 (6.26 ± 5) in the A-D measurement, followed by B-E (0–24(5.51 ± 4)) and A-C (0–15(4.27 ± 3)) measurements. No statistical difference was found between these values and age, gender, tooth position and procedure time.

The OHIP-14 and OHQoL-UK scales are the most preferred scales to evaluate the quality of life related to oral and dental health. The biggest advantage of OHIP-14 is that the questions are formed as a result of conversations with representative patient groups, not by the researchers, and the functional, psychological and social effects of oral cavity problems are determined by the patients [22]. Shugars et al. [23] used OHIP-14 to measure the patient’s perception of their experience after impacted third molar tooth extraction and found that patients may experience some symptoms and activity limitation for five days or less after surgery. Mcgrath et al. [11] In their study with OHIP-14 scores, they showed that quality of life was affected in the immediate postoperative period following the surgery of the impacted third molar. In our study, OHIP-14 was used to evaluate the postoperative quality of life of the patients. The OHIP-14 total score was calculated as 13.3 ± 8.19. It was observed that there was a negative correlation between OHIP-14 scores and patients’ gender, tooth position, duration of the procedure, alveolar osteitis, total pain, total swelling, difference AC, difference BE, difference AD scores, but none of these results were statistically significant. It was observed that there was a statistically significant relationship between the age of the patients and the difference mouth opening scores and the OHIP-14 scores. Based on these results, it was concluded that older patients and patients who developed trismus postoperatively were more affected functionally and socially in their lives.

### Limitation

Limited sample size, lack of demographic homogeneous structure, subjective forms and limited follow-up period are the limitations of the study. It can also provide more information about possible complications and imaging in evaluations using CBCT.

## Conclusion

As a result, considering that the incidence of impacted teeth has increased and impacted tooth surgery is the most common procedure in the field of maxillofacial surgery, it is of great importance to investigate the factors affecting complications and to minimize these complications, to put the right indication and to minimize the procedure time with the right surgical approach.

## Data Availability

The datasets used and/or analysed during the current study are available from the corresponding author on reasonable request.

## Abbreviations

OHIP: Oral health-related quality of life
VRS: Verbal rating scale
